# Challenges to Open-Heart Surgery in Sub-Saharan Africa: A Narrative Review

**DOI:** 10.21470/1678-9741-2024-0351

**Published:** 2025-10-31

**Authors:** Victory Bassey Effiom, Abdullah K. Alassiri, Victor Femi-Lawal, Eben-ezer Genda, Jonas Lotanna Ibekwe, Achanga Bill-Smith Anyinkeng, Olalekan Kolawole Victor, Echieh C. Peter

**Affiliations:** 1 Faculty of Clinical Sciences, University of Calabar, Calabar, Cross River, Nigeria; 2 Research Department, Association of Future African Cardiothoracic and Vascular Surgeons, Yaoundé, Cameroon; 3 Faculty of Medicine, King Abdulaziz University, Jeddah, Saudi Arabia; 4 Faculty of Clinical Sciences, University of Ibadan, Oyo State, Nigeria; 5 Faculty of Health Sciences, Official University of Bukavu, Bukavu, Democratic Republic of Congo; 6 Faculty of Health Sciences, University of Buea, Buea, Cameroon; 7 Division of Cardiothoracic Surgery, Department of Surgery, University of Calabar, Calabar, Cross River, Nigeria

**Keywords:** Open-Heart Surgery, Cardiac Surgery, Challenges, Africa

## Abstract

The rising cardiovascular disease burden in Africa necessitates a strengthened healthcare system including enhanced access to cardiac surgery, the definitive treatment for several surgical cardiovascular diseases. Though open-heart surgery, the most invasive type of cardiac surgery, was already possible in Africa over five decades ago, with pioneering surgeons performing atrial septal defect repairs via surface cooling in Ghana as early as 1964, its development across the continent has been hindered by significant challenges. This study highlights the challenges faced by both established and nascent open-heart surgery programs across Africa. We further identify key areas for sustaining and expanding open-heart surgery programs, including robust training for surgeons and support staff, resource allocation, and enhanced capacity building. By systematically analyzing the landscape of open-heart surgery in Africa, this paper proposes a multifactorial approach to overcome these limitations and ensure equitable access to this life-saving intervention for a vastly underserved population.

## INTRODUCTION

**Table:** 

Abbreviations, Acronyms & Symbols	
CCS	= Cardiac Center of Shisong
CVD	= Cardiovascular diseases
GNP	= Gross National Product
NCTC	= National Cardiothoracic Centre
OHS	= Open-heart surgery
PASCaTS	= Pan-African Society for Cardiothoracic Surgery

The journey of open-heart surgery (OHS) in Africa stretches back over five decades, with pioneering cardiac surgeons operating on atrial septal defects through surface cooling in Ghana as early as 1964. Notably, Nigerian patients did not benefit from such surgeries until much later, after 1974^[1]^. The establishment of independent cardiothoracic surgical centers could not take place before 1989 in Ghana while most African countries still lack sustainable cardiac surgery programs^[2,3]^. Since then, efforts towards the provision of cardiothoracic surgery services in teaching hospitals and advanced training of medical staff abroad have contributed some benefit to the well-being of cardiovascular diseases (CVD) patients individually and to the expertise of medical staff; however, morbidity and mortality rates remain high due to insufficient funding, poor equipment, and lack of skill transfer^[3]^.

The challenges to OHS in Africa can be categorized as political and apolitical. The political challenges include armed conflicts, diversion of fewer resources to prevalent infectious/communicable diseases such as malaria, acquired immunodeficiency syndrome (or AIDS), and enteropathies, and lack of internal funding on the part of governments as well as lack of foreign aid by the World Health Organization and other welfare organizations particularly for cardiac surgery^[4]^. Armed conflicts hinder foreign support for proctoring and divert investment to emergency trauma. The need to control the spread of prevalent communicable diseases and the relatively higher per capita cost of cardiac surgery programs reduces the prioritization of cardiac surgery by the government^[5]^.

The apolitical challenges to OHS are not unrelated to political decisions. In several African countries, the available medical facilities and expertise do not meet the prevalent needs^[5]^. Lack of cutting-edge surgical equipment or skill, fewer experts, and negligence towards the training of staff add to the issue^[2,3]^. Under-reported morbidity data and lack of research-based community engagement contribute to the prevalent suboptimal clinical care and neglect of policymakers^[6]^.

Although previous literature highlights the challenges of OHS in Africa while debating the need and ways to combat them^[4,5]^, most of the literature focused on the role of OHS in reducing morbidity and/or mortality in cardiac patients^[7-9]^ or was limited to institutional or regional peculiarities^[2,6,10]^. This narrative review demonstrates the most recent challenges as well as the overall condition of OHS in the entire continent and is thus aimed at filling the gap for a multifactorial approach towards this key issue.

## AN OVERVIEW OF AFRICA’S GEOGRAPHY, ECONOMY, AND DEMOGRAPHICS

Africa, a continent with diverse people, has increased its population to approximately 1.5 billion (approximately, 740 million women and 739 million men) inhabitants over the years, making it the second-largest continent after Asia^[11]^. The population of Africa over the years has been on a steady increase above 2.3% since 2000 and is expected to reach 2.5 billion by 2050^[12]^. The median age as of 2024, is 19.2 years, with approximately four live births per woman. The life expectancy in Africa for both sexes is 64 and independently, 66.1 years for females, and 62 years for males.

Its healthcare structure is still devastating, with over 43.3 infant deaths per 1,000 live births and over 62.4 deaths under five years per 1,000 live births, most likely because over 44% of its population is urban, and access to healthcare facilities and personnel it's still not accessible by the majority of its population^[13]^.

Africa's economy is estimated to have a gross domestic product of 2.6 trillion dollars considering its 54 member countries, with the largest economies being Nigeria, South Africa, Algeria, and Egypt^[3]^. It is also expected to reach 4.1 trillion dollars by 2027. Most of its income is accessed through agriculture, fishing, mining, and drilling primarily^[14]^.

## EARLY BEGINNING OF OPEN-HEART SURGERY IN AFRICA

The early beginnings of OHS in Africa were met with unique challenges and triumphs. As the field of cardiac surgery progressed globally, Africa grappled with limited resources, infrastructure, and expertise in this specialized area. Despite these challenges, several pioneering efforts and collaborations paved the way for the development of OHS in various African countries as discussed below.

Cardiothoracic surgery in Africa started as far back as 1940 with the establishment of thoracic surgery in South Africa, as a sub-division of general surgery at the time. Following the return of Professor Christiaan Barnard and Professor Rodney Hewitson from Minneapolis and London, respectively, the cardiothoracic unit in Cape Town, South Africa, was opened in 1958^[15]^. Nine years later, the first heart transplant was performed, leading to public awareness and advocacy for cardiac surgery, which brought in experts like Professor Robert Frater, Sir Terence English, and Professor Francois Hitchcock^[15,16]^. Professors Christiaan Barnard and Professor Rodney Hewitson played a major role in this public awareness and advocacy for cardiac surgery. Amazing work was done in pediatric cardiac surgery with the surgical treatment of tetralogy of Fallot, ventricular septal defect, and management of valvular disease through the designing of two mechanical valvular prostheses and Ebstein’s anomalies^[2]^. Shortly after Dr. Joe De Nobrego qualified, Professor Barnard retired, and the unit was manned by Professor Bruno Reichard^[15]^.

More African countries began to focus on building capacity for OHS within their healthcare systems. In West Africa, there has been a concerted effort by most countries like Ghana, Senegal, Nigeria, and Ivory Coast. In Ghana, the early start of cardiothoracic surgery could be dated back to 1964, when Professor Charles Odamtten Easmon’s team performed the closure of an atrial septal defect successfully using surface cooling to attain hypothermia, however, the efforts were halted after some time^[2]^. Following the return of Professor Kwabena Frimpong-Boateng in 1989, he braced the odds by establishing the National Cardiothoracic Center in Ghana at the Korle Bu Teaching Hospital in 1989; the center was officially commissioned on April 10^th^, 1992^[2]^. In Nigeria, cardiothoracic surgery started as far back as 1964, in Ibadan, following the establishment of the cardiac registry, and the first OHS was performed on February 1^st^, 1974, at the University of Nigeria Teaching Hospital in Enugu^[17]^ by a team of surgeons consisting of M. Yacoub, F.A. Udekwu, D.C. Nwafor, and C.H. Anyanwu^[2]^. This team of surgeons was also involved in attracting international collaborations and non-governmental organizations to ensure sustainability of OHS in Nigeria

In Ivory Coast, Professor H. Merle and Professor Yangni-Angate, who was a chief general surgeon, went to Houston (Texas, United States of America) for OHS training (congenital and pediatrics heart diseases and acquired heart diseases) under the mentorship of Professor Michael Debakey and Professor Danton Cooley; they pioneered the commencement of cardiothoracic surgery by performing the country’s surgical procedures at the Treichville Hospital in Abidjan^[4]^. Following the return of Professor Yangni-Angate, he was joined by Professor Metras (from France), Dr. Ouattara, and Professor Ouezzin-Coulibaly, who were cardiovascular and thoracic surgeons at Treichville Teaching Hospital. They performed the first OHS in the country on March 11^th^, 1978^[4]^.

In Senegal, Professor M. Ngiaye’s team at the Thoracic and Cardiovascular Surgery Department of Dakar’s Fann University Teaching Hospital commenced efforts towards an open-heart program in 1990^[2]^ in collaboration with non-governmental organizations. Prof. Ngiaye attracted non- governmental organizations and national and international collaborations to ensure sustainability of the training. In 1995, the first OHS occurred in Senegal, and till date they have grown to increase capacity in OHS^[17]^.

In 1989, cardiac surgery started in Zimbabwe, and from 1989 to 1992, they did 91 OHS having a mortality rate of 8.7%; just the locally trained cardiac surgeons performed over 400 OHS from 1995 to 2003, but due to sociopolitical scandals in the country, the OHS was suspended until February 2016 following donation of heart-lung machines from Medtronic to Parirenyatwa Group of Hospitals in Harare^[18]^. Cardiac surgery has also been reported in Kenya and Uganda. Most countries in East Africa have limited or no data about the early status of cardiothoracic surgery within the different countries.

Cameroon stands out and braced the odds to begin this surgical practice within Central Africa. The Cardiac Center of Shisong (CCS) located in the Northwest Region of the country was the lone cardiac surgical center in the country, brought to reality by the joint initiative of Bambini Cardiopatici nel Mondo and Cuore Fratello and local religious congregation of Tertiary Sisters of Saint Francis^[19]^. In November 2009, the CCS was the lone cardio-surgical center in the whole Central African Economic and Monetary Community, with an average number of inhabitants estimated to be 40 million. This led to the training of local surgeons to assist the Italian surgical team. From the time of creation, a total of 847 OHS have been performed, but due to the sociopolitical states between the government and separatist fighters, there was a need for relocation to the Outreach CCS located in Yaoundé, and surgical activities only resumed back in late 2019, being halted from 2018 to 2019^[20]^. Following a tweet from Dr. Manounda Malachie, the current Minister of Public Health of Cameroon, on the October 1st, 2022, congratulated the General Hospital Yaoundé that operated on four patients, aged 19 months and nine, 14, and 19 years, being the entire team made up of Cameroonians^[20]^. Again, Dr. Manounda Malachie tweeted on the October 26^th^, 2022, about the first coronary artery bypass surgery performed at Douala General Hospital, which was the sub-regional first of its kind^[21]^.

**Fig. 1:**
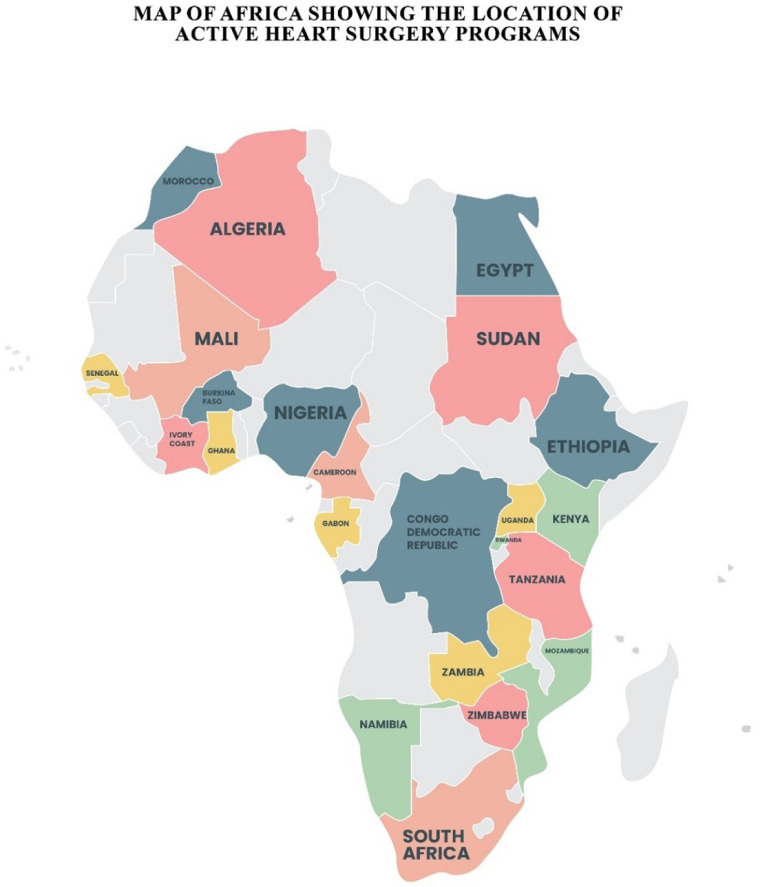
Map of Africa showing the location of active heart surgery programs.

Notable success stories, as seen in Table 1 and Figure 1 have emerged from other countries such as Egypt, Ethiopia, and Kenya, where dedicated medical professionals have been at the forefront of advancing cardiac surgical capabilities. Despite initial challenges, African cardiac surgeons and healthcare professionals demonstrated resilience and innovation in adapting OHS techniques to suit the local context.

**Table 1:** Healthcare characteristics in African countries.

S/N	Country	Population (millions)	Number of hospitals	Number of heart surgery centers	Number of open-heart surgeries per year	Type of resources
1	Cameroon^[25]^	28.6	-	3	39-50	Private/Public
2	South Africa^[25]^	60.41	Over 600	27	8400	Private/Public
3	Nigeria^[25]^	223.8	39914	15	100	Private/Public
4	Egypt^[25]^	112.7	1798	15	1000+	Private/Public
5	Ghana^[25]^	34.12	-	10	300+	Private/Public
6	Congo-Kinshasha^[25]^	102.3	-	2	-	Public/Private
7	Zambia^[25]^	20.57	-	2	-	Public
8	Uganda^[25]^	48.58	Around 150	5	-	Public
9	Kenya^[25]^	55.1	12,375	7	-	Private/Public
10	Zimbabwe^[25]^	16.67	214	1	-	Public
11	Senegal^[25]^	17.76	-	7	-	Private/Public
12	Burkina Faso^[25]^	23.25	-	3	-	Private/Public
13	Ivory Coast^[25]^	28.87	-	2	-	Public
14	Rwanda^[25]^	14.09	57	1	-	Public
15	Namibia[	2.694	36	1	280	Public/Private
16	Mali^[25]^	23.29	-	2	-	Public
17	Tanzania^[25]^	67.44	336	4	-	Public/Private
18	Ethiopia^[25]^	126.5	396	4	-	Public
19	Algeria^[25]^	45.61	297	18	7300	Private/Public
20	Mozambique^[25]^	33.9	-	2+	122	Public
21	Sudan^[25]^	48.11	438	5	-	Private/Public
22	Morocco^[25]^	37.84	Over 150	11	3500	Private/Public
23	Tunisia^[25]^	12.46	180	10	3000	Private/Public
24	Gabon^[25]^	2.437	-	2	-	Public
25	Eritrea^[25]^	3.749	22	1	-	Public
26	Madagascar^[25]^	30.33	125	-	-	Public/Private
27	Niger^[25]^	27.2	40	-	-	Public/Private
28	Angola^[25]^	36.68	-	-	-	Public/Private
29	Chad^[25]^	18.28	-	-	-	Public/Private
30	Benin^[25]^	13.71	-	-	-	Public/Private
31	Libya^[25]^	6.88	97	-	-	Public
32	Togo^[25]^	9.054	-	-	-	Public/Private
33	Somalia^[25]^	18.14	74	-	-	Public/Private
34	Malawi^[25]^	20.93	-	-	-	-
35	Togo^[25]^	9.054	-	-	-	Public/Private
36	Mauritania^[25]^	4.863	18	-	-	Public
37	Liberia^[25]^	5.418	39	-	-	Public/Private
38	Botswana^[25]^	2.675	26	-	-	Public
39	Central African Republic^[25]^	5.742	-	-	-	-
40	Seychelles^[25]^	119,773 (thousand)	6	-	-	Public
41	Guinea^[25]^	14.19	35	-	-	Public
42	Mauritius^[25]^	1.261	32	-	-	Public/Private
43	The Gambia^[25]^	2.773	-	-	-	Public/Private
44	Comoros^[25]^	852,075 (thousand)	15	-	-	Public
45	Djibouti^[25]^	1.136	16	-	-	Public/Private
46	Cabo Verde^[25]^	598,682 (thousand)	6	-	-	Public
47	Burundi^[25]^	13.24	109	-	-	Public/Private
48	São Tomé and Príncipe^[25]^	231,856 (thousand)	2	-	-	Public
49	Guinea-Bissau	2.151	8	-	-	Public
50	Equatorial Guinea^[25]^	1.715	18	-	-	Public
51	Senegal^[25]^	17.76	38	-	-	Public
52	Lesotho^[25]^	2.33	18	-	-	Public/Private
53	Eswatini^[25]^	1.211	14	-	-	Public/Private
54	Sierra Leone^[25]^	8.791	80	-	-	Public/Private

## CHALLENGES

### Low Volume of Cases

There are fewer thoracic surgery centers and institutions on the continent than there should be. Nevertheless, the number of open-heart operations per facility is low, despite the crying need for these procedures in the general population^[22]^. The inability to pay for this surgery explains this imbalance between the need and the number of procedures performed in the cardiothoracic surgery center^[4,23]^.

In some centers, there are long periods during which no OHS is performed. Operating on certain cases can appear difficult in certain centers, which motivates transfers outside the continent and thus significantly reduces the average number of cases operated on locally^[7]^.

The lack of surgical materials and prostheses is a typical example of the reasons why patients must wait or be transferred^[6]^. The small number of cardiothoracic surgeries and cardiothoracic surgery centers also poses a problem.

### Training and Exposure

Raising trends for CVDs with increased hospitalizations and greater demand for surgical intervention requires a higher number of trained cardiothoracic surgeons and supporting staff. But despite efforts of decades and multidisciplinary actions, none of the African countries succeeded in the desirable training of cardiac surgeons both in numbers and skill^[3]^.

The sharply increasing burden of CVDs led to the development of various training programs for African cardiac surgeons. There are several notable training programs, like the Pan-African Society for Cardiothoracic Surgery (PASCaTS), which is a platform for advanced cardiothoracic surgical training of African medics. The PASCaTS project led by Charles Yankah has managed to keep the training channel open through webinars and is currently seeking on-site mentorship^[24,25]^.

Government-funded or self-funded training of African cardiologists in developed countries has also benefited the local population^[6]^. But keeping in view the enormity of the CVD burden, the gravity of socioeconomic and political conditions, and the population needs, these training programs require funding on a larger scale.

### Laboratory Support Facilities (Radiology and Blood Bank Facilities)

To achieve a successful OHS, the need for 24-hour laboratory support for radiological imaging and blood supply cannot be overemphasized^[26,27]^. In Africa, access to this laboratory support has varied significantly over the years, with more improvement becoming obtainable in recent times. In Nigeria, for example, at the Lagos State University Teaching Hospital, laboratory support for full blood counts, electrolytes, and liver function tests, and clotting profiles is usually obtainable during working hours. Blood products comprising fresh frozen plasma, platelets, and cryoprecipitate can equally be obtained; however, oftentimes, delay arises between time of request and delivery. Sometimes, there is a lack of blood in the blood bank due to the unwillingness of individuals to donate blood at the hematology department. Due to this, strenuous efforts are often made to minimize the risk of postoperative bleeding with the use of antifibrinolytics. Also, the unavailability of potent broad-spectrum antibiotics is sometimes a challenge that has been worsened by the depressed economy in several countries and the high resource consumption of performing OHS^[26]^. In the same vein, the diagnosis of several CVDs in Africa is often reliant upon the use of radiological imaging such as X-ray, computed tomography scan, ultrasound scan, and echocardiography. Only a few referral centers in urban areas perform these procedures due to the lack of human resources for cardiovascular care.

Therefore, patients often end up going to these few centers, sometimes far away from their locality, to obtain the necessary imaging required for any advancement in their care. In most countries of sub-Saharan Africa, interventional cardiology and cardiac surgery are not obtainable; therefore, some cardiac centers rely on collaborative partnerships which often involve the referral of patients to these partnering centers^[26,28]^.

### Cardiac Catheterization

Cardiac catheterization is a key investigation for the diagnosis and treatment of CVD, especially coronary artery disease. It is one of the most commonly performed procedures with the highest volume seen in the United States of America where over a million coronary angiograms were performed as of 2011. Ever since the first performance of right heart catheterization by Grossman in 1929, cardiac catheterization has evolved significantly. In 1959 and 1977, Stones and Gruntzig performed selective coronary angiography and coronary balloon angioplasty, respectively. Today, it has evolved into diagnostic and interventional techniques used for repairing septal and valvular defects^[29]^. However, in sub-Saharan Africa, there is a massive shortage of cardiac catheterization centers, and most countries do not meet up with the minimum recommended standard of one center to 1,000,000 population^[28,30]^. As of 2018, just 38 cardiac catheterization centers existed in sub-Saharan Africa. In West Africa, 13 centers existed, six in Nigeria, four in Senegal, two in Ghana, and one in Cameroon. East Africa had five centers in Kenya, three in Ethiopia, two in Tanzania, and one in Uganda. Furthermore, Mauritius had six centers, Namibia had two, and Madagascar and Botswana had one each. Angola and Mozambique had four and two centers, respectively. Cape town, South Africa, currently has 13 cardiac catheterization centers, and Johannesburg, still in South Africa, also has at least 13 cardiac catheterization centers. Oftentimes, the high cost of installing and maintaining such centers, the need for trained and dedicated personnel, and the need to continually stock a large inventory of expensive consumables for diagnoses and interventions are the limiting factors for setting up these centers^[28]^. Therefore, collaborative partnerships are frequently established to meet the needs of patients either by bringing in a team of experts to assist with cardiac catheterization or referring patients to such centers^[26,29]^.

### Financial Support

OHS is an expensive surgery. This is so because high-level technology and infrastructure, which require high cost to maintain, are needed. This is worsened by the fact that this equipment must be imported to African countries. The exchange rate of dollars to African countries' currencies keeps increasing, and this escalates the cost of OHS in African countries^[30]^. For instance, consumables for bypass and pharmaceutical agents needed for the surgery must be imported from other countries^[18]^. Also, a continuous supply of electricity is needed in a center that will major in cardiac surgery, such as the OHS. Most of Africa doesn't have a constant electricity supply. This means that to maintain OHS, the hospital will have to get an alternative power supply. This will further increase the financial implications of performing OHS.

Citizens of most African countries cannot pay for OHS, and there is no health insurance scheme in most of these countries. For those who are on a health insurance scheme, it does not cover the cost of OHS. In Nigeria, the National Health Insurance Scheme does not cover OHS^[18]^. In Zimbabwe, < 10% of the population subscribes to health insurance companies^[18]^. As of 2011, the gross cost of OHS in Nigeria was between the range of US$6,230 and US$11,200^[19]^. US$5,000 was the cost of OHS for Ghanaians at the National Cardiothoracic Centre in Ghana. This is so because a 50% subsidy of the cost is paid by the Ghanaian Heart Foundation^[23]^. In Kenya, there is a form of subsidy on OHS by the government to the citizens^[23]^. These amounts show that it is cheaper to get OHS in one's country compared to if a patient chooses the option of getting OHS done abroad. However, most Africans due to the economic situation of the region cannot afford the cost of the surgery. The other options that African patients who need OHS often receive are donations and looking for sponsors.

### Moving from the Cardiac Mission Model

The cardiac mission model has been the mainstay of open cardiac procedures, particularly in the low-resource settings of African countries. It has been critical as a temporary salve in the underdeveloped world, where there is significant mortality from congenital heart diseases that are resolvable with OHS^[31]^. In some areas, these short-term surgical missions are still the only resource available, highlighting their essence.

Cardiac missions can be relatively cost-effective despite long-held misperceptions. In a paper published by Polivenok I et al.^[31]^, the authors established that individuals who have the opportunity to benefit from these missions experience improved life expectancy, and the benefits also reflect on society.

However, significant challenges exist with sustainability due to the dependence of this model on external expertise. Often, patients must either leave their native countries for procedures or wait long periods for foreign-assisted missions. Additionally, the costs of unmet needs indicate the need for effective transition. In the case of Ghana, average costs per patient for cardiac surgery abroad were as high as US$50,000 in the 1990s, excluding other paraphernalia. Comparatively, average costs for procedures performed locally were in the range of US$6,000 per patient at the same period^[17,32]^.

It is also important to note that these few cardiac missions are insufficient to cope with the significant burden of diseases in the region. The highly cited Cape Town Declaration highlights this. It reveals that Africa’s over one billion people have access to only 22 cardiac centers, with the remaining burden on cardiac missions^[33]^. The relative sparseness of existing cardiac missions indicates that it is essential for independent OHS practice in Africa to thrive.

The challenge of moving from the cardiac mission model is primarily economic. Edwin et al.’s^[2]^ study compared the Gross National Products (GNPs) of areas with a high concentration of cardiothoracic surgeons to those with fewer, finding that the density of cardiothoracic surgeons is strongly linked to GNP, likely due to the financial input required to establish an OHS practice and the costs to the patients. Without a guarantee of a viable model for meeting the associated healthcare costs, it becomes a significant burden for governments to bear the expenses, particularly in the context of other infectious diseases, which remain the priority of African healthcare systems^[18]^.

In Senegal, a collaboration between several non-governmental organizations and arduous efforts at the Fann University Teaching Hospital resulted in an OHS program. The first case involved a large team from the United States of America at Dakar’s Aristide Le Dantec Hospital; currently, Senegal independently offers OHS^[2,34]^. In moving from the cardiac mission model, it is important to ensure that original solutions are proffered for unique challenges. Ghana, Ivory Coast, and Senegal have had to surmount several challenges in sustaining their programs, and these represent a model that many African countries may emulate in developing and sustaining their own programs.

## FUTURE DIRECTIONS FOR OPEN-HEART SURGERY IN AFRICA

It is demonstrable that sustainable OHS is possible in Africa, as has been indicated in the cases of Ivory Coast, Ghana, and Senegal. The need for OHS is also evident for both economic and health reasons^[2,18,31]^. Based on the challenges that recur across Africa’s (attempted and successful) OHS programs, the authors propose several areas of focus.

A key priority for improved outcomes is a national commitment to strong cardiac surgery programs. The cases of Senegal and Ghana are as much a reflection of national commitments as they are of the work of passionate professionals and sturdy institutions. Cardiac surgery is expensive, requiring dedicated theatres, intensive care units, cardiac catheterization, radiology, echocardiography, renal dialysis units, and other ancillary facilities. Thus, there is a need for sustained economic input, which can be more readily facilitated by governments and their instruments.

African governments can play an important role in addressing disparities in cardiac care across the continent. The government can prioritize healthcare funding by ensuring that medical facilities are equipped with the necessary technology and resources to diagnose and treat heart conditions effectively. Furthermore, they should implement policies that support healthcare worker training and retention so as to enhance the quality of cardiac care. Moreso, the governments should invest in public health campaigns to raise awareness about heart disease prevention and early detection, empowering citizens to take proactive steps towards their cardiac health. Establishing national registries and databases is another area the governments can look into as this would better monitor heart disease trends and outcomes, allowing for targeted interventions.

International collaboration, within and outside Africa, is also crucial. International organizations can provide funding for healthcare infrastructure, ensuring that medical facilities are adequately equipped to diagnose and treat heart conditions. They can facilitate training programs, enhancing the skills of the cardiac surgeons within the continent, enabling them to deliver high-quality cardiac care. Additionally, these organizations can promote research collaborations, leading to the development of region-specific treatments and interventions. Advocacy and awareness campaigns spearheaded by these organizations can also educate the public about cardiac conditions, encouraging preventative measures and early detection.

The National Cardiothoracic Centre (NCTC), Ghana, has been accredited as a center of excellence for training cardiothoracic surgeons by the West African College of Surgeons. Since its accreditation, Edwin and Frimpong-Boateng write that over 20 surgeons from Nigeria, Togo, and Ethiopia have trained at the center^[17]^. Centers such as the NCTC have been of great help in Africa’s cardiac surgical mission and need to be replicated across the continent, and such a replication would need to be supported by the home countries of these programs. A recent analysis reveals Africa contributes to only 3% of the global cardiovascular research, despite its significant disease burden. Therefore, the development of research capacity is a major need^[35]^.

The establishment and standardization of training should be a critical aspect of local and international efforts. In the Cape Town Declaration, Zilla et al.,2018^[33]^ recommend an international working group that would develop criteria for standard cardiothoracic surgery training programs in Africa^[29]^. This is critical for the context of low and middle-income countries, as Western training programs may lack the pathologies and setups required to operate in this region. International collaboration and the raising of capital are also required in establishing centers of excellence, for which unmet needs exist: all sub-Saharan Africa has access to only 22 cardiac centers^[36]^.

While building Africa’s OHS programs, it is important to ensure that things grow on a solid base. This specifically refers to the cardiac mission model. While indigenous programs are being implemented, it is important to maximize the existing support of cardiac missions. This approach has worked in multiple instances, albeit with local resourcefulness and commitment. In Lagos State University Teaching Hospital, for example, three separate cardiac missions were carried out between 2004 and 2006. The program was a collaboration between the hospital and Global Eagle Foundation, based in the United States of America. In this case, major training and collaboration were organized before and after the missions, enabling the development of local capacity. It was successful, and surgeons at the center now regularly perform elective cardiac surgery^[37]^.

Without support from other stakeholders, the efforts of public institutions alone may not be sufficient. Therefore, in addition to national efforts, it is important to support moves by private institutions, which can improve access significantly. The efforts of surgeons and administrators at Biket Medical Centre, a privately-owned hospital in Osogbo, Nigeria, are worthy of mention here. In initial reports of OHS outcomes, the center operated on a hybrid model with efforts from local surgeons and an expatriate team from Israel and India. In a year, the team performed 24 cardiac surgeries, becoming the first private hospital to do so in West Africa^[38]^. With adequate support from private initiatives, it is possible to improve outcomes significantly.

While the need for OHS is undeniable, many patients have stalled due to a lack of funds to afford the service. Therefore, the cost of an OHS remains a significant burden on patients. Specialized equipment and consumables such as heart-lung machines, X-rays, and medications are not produced locally, limiting affordability and access. In Zimbabwe, the repair of septal defects typically costs between US$4,000 and US$6,000^[18]^. While this is far cheaper than what is obtained in America and South Africa, it remains a burden for a significant population. Nigeria, Africa’s wealthiest country, has a gross national income per capita of only US$2,140. It is far lower in countries such as Niger, with only $610^[39]^. Machawira et al.^[18]^ suggest that African cardiac centers try to buy consumables in bulk — but this is only possible if turnover rates are compatible. Each country will need to assess its realities and devise a sustainable payment plan for its citizens — ideally, one that incorporates a robust insurance scheme. Finally, African cardiac surgeons who are trained outside of Africa should consider secular migration as a means of skilled transfer and domestic skill development. This approach will help in low-volume centers and efforts must remain sustained, as success will take years of focused work.

## CONCLUSION

The need for sustainable local cardiac surgery centers that can perform OHS in African nations cannot be denied. With adequate willpower, favorable policy, and good support from all the stakeholders and the masses, this will be achieved. The success story of the very few African countries as regards OHS can be adopted, modified, and restructured to suit the peculiarities of each African nation. Furthermore, there should be reliable and sustainable efforts by the government to aid their citizens who need OHS. This could be in the form of loans, subsidized surgery, or a health insurance scheme that covers OHS. Governments of the different African countries should ensure compliance with the Abuja Declaration on health. The Abuja Declaration was adopted by the African Union governments on April 27th, 2001, with the sole aim of setting a target of allocating at least 15% of each government national budget to improve health care. Ensuring compliance with the Abuja Declaration will help to make cardiac surgery sustainable to the population. Lastly, private insurance can also be encouraged to have a scheme that focuses on cardiac surgeries. This will make it easier for people to be able to afford OHS.

## Data Availability

The authors declare that the data supporting the findings of this study are available within the article.
